# Deciphering *Trichoderma viride*-mediated cadmium stress alleviation in wheat: morphological, physiological and biochemical insights

**DOI:** 10.1186/s12870-026-08621-8

**Published:** 2026-04-13

**Authors:** Rabab A. Metwally, Reda E. Abdelhameed

**Affiliations:** Botany and Microbiology Department, Faculty of Science, Zagazig University, Zagazig, 44519 Egypt

**Keywords:** Cadmium toxicity, *Trichoderma viride* RA1, *Triticum aestivum*, Antioxidant capacity, Osmoprotectants, Bio-accumulation, Lipid peroxidation

## Abstract

**Background:**

The extremely hazardous metal cadmium (Cd) restricts plant growth and interferes with many morphological, biochemical and physiological functions. One biotechnology strategy is the application of fungi to eliminate harmful pollutants from the environment.

**Methodology:**

*Trichoderma viride* strain RA1 is employed to investigate its prospective for Cd scavenging and promoting wheat (*Triticum aestivum* L.) growth. The colony diameter and mycelial dry weights (dwt) of *T. viride* were examined. Also, a randomized pot experiment was conducted to mitigate the Cd harmful effects in wheat plants using *T. viride* RA1grown under Cd-stressed (200 mg/L) conditions.

**Results:**

The results revealed that with increasing Cd conc., colony diameter and mycelial dwt of *T. viride* decreased. Also, various phenotypic, physiological and biochemical characteristics were evaluated. Cd content in *T. aestivum* shoots and roots were appraised. Regarding osmoprotectants, the highest increase in proline content (16.14%), glycine betaine (24.95%) and protein (28.07%) was detected in *T. aestivum* plants with *T. viride* RA1 beneath Cd imposition. The MP-AES results indicated a greater buildup of Cd in the roots of plants inoculated with *T. viride* RA1 than non-inoculated ones. Our data suggest that *T. viride* can be utilized to increase total antioxidant capacity and osmoprotectant levels while decreasing malondialdehyde, electrolyte leakage, in addition to H_2_O_2_ levels in order to lessen the detrimental effects of Cd on *T. aestivum* plants.

**Conclusion:**

According to our findings, *T. viride* RA1 may function as a bio inoculant, encouraging *T. aestivum* development under Cd stress, thereby assisting sustainable farming methods.

## Key points

- Increasing Cd concentration led to a decrement in colony diameter and mycelial dry weight of *T. viride*.
- Cd adversely affects *T. aestivum* growth and causes comparable changes in its physio-biochemical responses.
- *T. viride* RA1 function as a bio inoculant, encouraging *T. aestivum* development in the presence of Cd stress.
- The growth traits and chlorophyll of *T. aestivum* treated with Cd were significantly increased by *T. viride* RA1 inoculation.
- *T. viride* RA1 contributed to the increase of antioxidant capacity and alleviated the stress markers in *T. aestivum.*

## Background

The discharge of heavy metal (HM) pollutants from several sources, including wastewater, industrial processes, agricultural chemicals, and metal processing has notable implications. The microbes and plants in the soil can be negatively impacted by these HMs, which have a cascading effect on soil characteristics and agricultural output [1–3]. Although some of them are micronutrients (i.e. Zn, Cu, Ni, and Mn, are beneficial at low concentration and become hazardous at larger amounts) and others are non-essential and very toxic to all organisms even at quite low levels [4, 5]. Cadmium (Cd), one of non-essential and toxic HMs, accumulates and intensifies in crops along the food chain when groundwater is used for irrigation, causing visual impairments. Furthermore, Cd reduces the effectiveness of transpiration and interferes with photosynthesis in plants. Its buildup in plants is determined by their genetic composition, and because it accumulates in soil, remediation techniques become difficult, making a sizable amount of agricultural land unusable for farming [3, 4].

However, array of methods, for instance ion exchanges, filtration, chemical precipitation, and evaporation, have been reported throughout decades to address this problem [4]. It is practically impossible and unfeasible economically to salvage HMs from polluted soil using these methods. These restriction prompted researchers to look into the use of microorganisms, such as fungi, as HM scavengers, economically viable, environmentally benign, and cost-effective [6, 7]. Also, the fungi have a high facility to produce extracellular degrading enzymes for eradicating toxic contaminants. Depending on the type as well as the HM concentration, the fungus has varying thresholds for its tolerance. The most likely cause of the variance in the fungal reaction to trace metals is intrinsic physiological variables [8]. Gupta et al. [6] stated that *Aspergillus niger* bio-absorbed Ni from the Ni-polluted effluents. Also, Cai et al. [9] reported the potential of *Mucor* sp., *A*. *niger* and *Pencillium chrysogenum* in Ni, Zn, Cd and Pb removal.

Up to 90% of soil processes are ascribed to soil microorganisms; without them, the soil would be lifeless [10, 11] because of their ability to interact with one another as well as with other living organisms such as plants [12, 13]. Re-establishing microorganism populations is essential to restoring the degraded soil because HMs-degraded sites frequently have poor microorganism activity [11, 14]. According to Asghar and Kataoka [15], rhizosphere fungi increase the root as well as shoot dry weights of tomatoes and *Arabidopsis thaliana* by exposing plants to diverse volatile organic compounds. Through processes similar to those of mycorrhizal fungi, the genus *Trichoderma*, a fungus that promotes plant growth, encompasses yield production, crop quality, as well as total growth [16]. It has been proven that a number of *Trichoderma* spp. can enrich plant growth [17–19]. Furthermore, it has been discovered that *T. atroviride* and *T. virens* form indole acetic acid besides other substances related to auxin, which is indispensable for root development in the early phases of plant growth [20]. Moreover, Anand et al. [21] stated that *T*. *viride* scavenges Cu through binding with cell wall. Moreover, *T. asperellum* and *Hypocrea nigricans* had the capacity to manage soil borne phytopathogens, promote plant growth and scavenge Cd as well as Pb [4]. Ng et al. [22] confirmed that the *T*. *reesei* had an efficacy on Cr (VI) adsorption. Moreover, *T*. *viride* and *T. asperellum* had the ability to enhance both morphological and physio-biochemical parameters of tomatoes and barely plants under salinity and pathogen attack [18, 19].

Since bread is considered to be the mainstay of the Egyptian diet, wheat (*Triticum aestivum* L.) is one of the most significant cereal crops. It is also a strategically important crop that provides an economic return for Egyptian farmers [23, 24]. Wheat, like other plants, is sensitive to a variety of biotic and abiotic stresses, including HMs [25, 26] which can negatively affect plant growth in numerous ways, both directly and indirectly [27]. Oxidative stress has been demonstrated to be induced by HMs through the generation of toxic reactive oxygen species (ROS), alteration of the antioxidant defense system, substitution of transcription factors and enzyme cofactors, cellular redox and ionic transport imbalance, damage to DNA and membranes, and oxidation of proteins [26, 28, 29].

Despite substantial evidence supporting the role of *Trichoderma* spp. in alleviating Cd toxicity in plants, several critical gaps remain. Most previous studies focus on mixed or other species of *Trichoderma*, with limited emphasis on species-specific mechanisms, particularly those mediated by *T. viride*. In wheat, one of the world’s most important staple crops, systematic investigations addressing *T. viride*–induced Cd stress mitigation are still scarce. As a result, the coordinated responses underlying *T. viride*–mediated Cd tolerance, including the interplay between photosynthetic efficiency, membrane integrity, osmolytes accumulation, and antioxidant defense, remain poorly understood. Therefore, a clear research gap exists in comprehensively elucidating the multi-level protective mechanisms of *T. viride* RA1 as a strain-specific efficacy, stress-adaptive potential and its first integrative evaluation in alleviating Cd stress in wheat under in vivo and in vitro studies. Addressing this gap will provide deeper mechanistic insights into how *T. viride* enhances wheat tolerance to Cd toxicity and will strengthen its potential application as a sustainable biological strategy for managing HM stress in agroecosystems. Therefore, this study investigated the Cd tolerance of *T. viride* RA1 and its potential to mitigate Cd toxicity in *T. aestivum* by regulating Cd accumulation, modulating plant growth and physio-biochemical responses under controlled and Cd-stressed conditions.

## Materials and methods

### Fungal inoculum preparation

*Trichoderma viride* strain RA1 was molecularly recognized and put in NCBI GenBank (ON479613). It was earlier obtained from El-Sharkia governorate soil rhizosphere. *T. viride* RA1 was cultivated on Potato dextrose-agar (PDA) (Sigma-Aldrich, St. Louis, MO, USA) medium for seven days at 27 ± 2 °C until sporulation [30].

## In vitro studies

### Effect of Cd conc. on radial growth of *T. viride* RA1

Active culture of *T. viride* RA1 mycelia discs (9 mm in diameter) were cultivated at 27 ± 2 °C after being planted on PDA media containing varying conc. of CdCl_2_ (2.5, 5, 10, 25, 50, 100, 200, 300, 400, and 500 mg/L). PDA media with *T. viride* RA1 mycelia disc but without CdCl_2_ (0 mg/L) served as the control. Five days following inoculation, a ruler was used to measure the diameter of each colony at its broadest section. Additionally, hyphal threads of *T. viride* RA1 fungal colony at its ends were examined for the morphological abnormalities generated by Cd on the mycelia of *T. viride* RA1under a light microscope in comparison to the control (Leitz WETZLAR, Germany).

### Effect of Cd conc. on mycelia dry weight, Cd removal and bioaccumulation efficiency of *T. viride* RA1

*T. viride* RA1 mycelial discs cultivated on PDA medium were moved to 250 mL Erlenmeyer flasks containing 100 mL of potato dextrose broth (PDB) media with different CdCl_2_ concentrations (2.5, 5, 10, 25, 50, 100, and 200 mg/L). The control group consisted of media without CdCl_2_ solution (0 mg/L). Flasks were kept in an incubator at 27 ± 2 °C for seven days. On day 7, the fungal mycelia were filtered through sterilized filters and washed 3 times with distilled water, dried for 2 days at 60 °C, and weighed on Whatman no. 1 filter paper (pre-weighed) to determine their dry weight (dwt). Additionally, culture broths that had been filtered were collected for Cd analysis by the Agilent 4210 MP-AES (Microwave Plasma Atomic Emission Spectrometer, Agilent Inc.) at the Ecology Laboratory, Faculty of Science, Helwan University. This instrument was fitted with a quartz torch, a glass concentric nebulizer, and a cyclonic spray chamber. The viewing position and nebulizer gas flow rate were optimized prior to each analytical run using the MP-AES system. Internal instrument calibration was performed using an aqueous calibration solution. Five aqueous calibration standards for each element were prepared in the ppm range by appropriate dilution of a commercial multi-element Merck IV standard solution. Instrument recovery ranged from 95 to 110%, depending on the element, in accordance with the manufacturer’s specifications. All measurements were conducted in triplicate.

Also, Cd removal (Re) and bioaccumulation efficiency (Q) of *T. viride* RA1 were calculated by difference from the initial solution concentration.

$$\:\mathbf{R}\mathbf{e}\:\left(\mathbf{\%}\right)\:=\frac{\:\mathbf{C}\mathbf{i}-\mathbf{C}\mathbf{f}\:}{\:\:\:\:\mathbf{C}\mathbf{i}\:}\:\mathbf{X}\:100$$

$$\:\mathbf{Q}\:=\frac{\:\mathbf{C}\mathbf{i}-\mathbf{C}\mathbf{f}\:\:}{\:\:\:\mathbf{m}\:}\:\mathbf{X}\:\mathbf{V}$$

Where, Re: removal efficiency (%); Q: bioaccumulation (metal uptake; mg/g dwt of fungal biomass) capacity, Ci and Cf are the initial and final conc. of Cd (mg L^− 1^); respectively, m is dwt of fungal biomass (g), and V (L) is the initial volume of aqueous medium.

### Cd tolerance index (TI) of *T. viride* RA1

The TI to Cd is acquired by dividing the growth of *T. viride* RA1 fungus exposed to different Cd conc. by its growth in the control medium [31].

$$\:\mathrm{T}\mathrm{I}\:=\frac{\:\mathrm{f}\mathrm{u}\mathrm{n}\mathrm{g}\mathrm{a}\mathrm{l}\:\mathrm{g}\mathrm{r}\mathrm{o}\mathrm{w}\mathrm{t}\mathrm{h}\:\mathrm{u}\mathrm{n}\mathrm{d}\mathrm{e}\mathrm{r}\:\mathrm{C}\mathrm{d}-\mathrm{t}\mathrm{r}\mathrm{e}\mathrm{a}\mathrm{t}\mathrm{e}\mathrm{d}}{\:\:\:\mathrm{f}\mathrm{u}\mathrm{n}\mathrm{g}\mathrm{a}\mathrm{l}\:\mathrm{g}\mathrm{r}\mathrm{o}\mathrm{w}\mathrm{t}\mathrm{h}\:\mathrm{u}\mathrm{n}\mathrm{d}\mathrm{e}\mathrm{r}\:\mathrm{c}\mathrm{o}\mathrm{n}\mathrm{t}\mathrm{r}\mathrm{o}\mathrm{l}\mathrm{l}\mathrm{e}\mathrm{d}\:\mathrm{c}\mathrm{o}\mathrm{n}\mathrm{d}\mathrm{i}\mathrm{t}\mathrm{i}\mathrm{o}\mathrm{n}\mathrm{s}}\:$$

### In vitro preliminary experiment on wheat under Cd stress

Wheat (*Triticum aestivum* L.) (Sakha 93) seeds were obtained after permission from the Department of Agricultural Research Center, Giza, Egypt, and their growth below various Cd conc. were examined in an in vitro experiment. The Agricultural Research Centre in Giza, Egypt, provided the wheat seeds. Inside laminar flow cabinet, seeds were surface-sterilized by adopting it in 30% (v/v) sodium hypochlorite solution (4 min) followed by repeated washing with dist. water and extra dried on autoclaved paper. Seeds were dispensed in jars containing water-agar (0.8%) with different Cd conc. (0, 5, 25, 75, 100, 150 and 200 mg/L). Jars were kept in an incubator under controlled conditions allowing the seeds to germinate. After 5 days, germination parameters such as germination rate, vigor index, seedling fresh weight (fwt), length and dwt were measured.

$$\:\mathrm{G}\mathrm{e}\mathrm{r}\mathrm{m}\mathrm{i}\mathrm{n}\mathrm{a}\mathrm{t}\mathrm{i}\mathrm{o}\mathrm{n}\:\mathrm{r}\mathrm{a}\mathrm{t}\mathrm{e}\:\left({\%}\right)=\frac{\:\mathrm{N}\mathrm{u}\mathrm{m}\mathrm{b}\mathrm{e}\mathrm{r}\:\mathrm{o}\mathrm{f}\:\mathrm{g}\mathrm{e}\mathrm{r}\mathrm{m}\mathrm{i}\mathrm{n}\mathrm{a}\mathrm{t}\mathrm{e}\mathrm{d}\:\mathrm{s}\mathrm{e}\mathrm{e}\mathrm{d}\mathrm{s}\:\:}{\:\:\:\mathrm{t}\mathrm{o}\mathrm{t}\mathrm{a}\mathrm{l}\:\mathrm{n}\mathrm{u}\mathrm{m}\mathrm{b}\mathrm{e}\mathrm{r}\:\mathrm{o}\mathrm{f}\:\mathrm{s}\mathrm{e}\mathrm{e}\mathrm{d}\mathrm{s}\:}\mathrm{X}\:100$$

$$\:\mathrm{V}\mathrm{i}\mathrm{g}\mathrm{o}\mathrm{r}\:\mathrm{i}\mathrm{n}\mathrm{d}\mathrm{e}\mathrm{x}\:=\left(\mathrm{M}\mathrm{e}\mathrm{a}\mathrm{n}\:\mathrm{s}\mathrm{e}\mathrm{e}\mathrm{d}\mathrm{l}\mathrm{i}\mathrm{n}\mathrm{g}\:\mathrm{l}\mathrm{e}\mathrm{n}\mathrm{g}\mathrm{t}\mathrm{h}\right)\:\times\:\:\mathrm{g}\mathrm{e}\mathrm{r}\mathrm{m}\mathrm{i}\mathrm{n}\mathrm{a}\mathrm{t}\mathrm{i}\mathrm{o}\mathrm{n}\:\mathrm{r}\mathrm{a}\mathrm{t}\mathrm{e}\:{\%}$$

## In vivo wheat growth under the effect of *T. viride* RA1 and Cd under greenhouse conditions

### Plant experimental conditions

Through conducting an experiment in the greenhouse of the Botany and Microbiology Department of Faculty of Science in Zagazig University, the bioremediation capacity of *T. viride* RA1 was ascertained. First, *T. viride* RA1 was cultivated for seven days at 25 ± 2 °C on PDA medium. Then spores were washed with water, diluted to 1 × 10^7^ cfu/mL and used to inoculate half of sterilized pots contained autoclaved soil at 121 °C for 60 min on three consecutive days and watered every 3 days for 10 days. Secondly, the surface sterilized *T. aestivum* seeds were sown in all pots. After 25 days of sowing, Cd (200 mg/L; this conc. was selected based on the preliminary experiment) was added to the soil. The soil had a pH of 7.7, clay texture (clay: 62.8%, silt: 25.5%, sand: 9.7%), saturation percent of 60%, electric conductivity of 1.93 dS m^− 1^, cation content of K^+^ = 0.42, Mg^2+^= 4.98, and Ca^2+^= 10.61 mEq L^− 1^ and anion content of SO_4_^2−^= 6.57, Cl^−^= 8.47, (HCO_3_)^−^= 5.66, (CO_3_)^−^= 0 mEq L^− 1^. Total N, P, and K are 0.58%, 0.66%, and 0.33%, respectively, and contain no Cd (0 mg kg^− 1^). There were four treatments as shown in Table 1 and five replicates were maintained for each treatment.

**Table 1:** The treatments used in the greenhouse experiment

Parameter Code	Treatment
Control	Non-inoculated and non-stressed *T. aestivum* plants
Cd (200 mg/L)	Non-inoculated and Cd-stressed *T. aestivum* plants
*T. viride* RA1	*T. viride* RA1-inoculated and non-stressed *T. aestivum* plants
*T. viride* RA1 + Cd	Cd-stressed and *T. viride* RA1-inoculated *T. aestivum* plants

### Growth parameters and photosynthetic pigments

After 30 days of Cd application, *T. aestivum* plants were harvested, and their roots were delicately cleaned with tap water before being dried on paper towels. The fwt of the plant roots and shoots were recorded. To ascertain their dwts, *T. aestivum* roots and shoots were dried independently in an oven for 72 h at 60 °C. Quantitative measurements of *T. aestivum* photosynthetic pigments were accomplished (carotenoids, chlorophyll a, and chlorophyll b) [32] after extraction with 85% acetone. At 663, 644, and 452 nm, absorbance was measured *via* a UV-visible spectrophotometer [RIGOL, Model Ultra-3660].

### Stress markers (membrane damage traits, malondialdehyde and hydrogen peroxide) in *T. aestivum* under Cd stress

Leaf membrane damage of *T. aestivum* upon Cd exposure was determined through electrolyte leakage [EL] [33]. Glass bottles containing 0.5 g of chopped *T. aestivum* leaves that had been divided into tiny (10 mm) segments were filled with 30 mL of deionized water. The bottles were incubated at 95 °C for 30 min to measure the final electrical conductivity (EC2) of the solution after being left at room temperature for 24 h to measure the solution’s initial EC1. Moreover, membrane stability index (MSI) of leaves was assessed. Additionally, the ratio of the MSI of *T. aestivum* under Cd stress to that of the controls was used to assess membrane injury (MI) [34]. The EL, MSI and MI were defined as follow:

$$\begin{array}{c} \mathrm{EL}\ (\%) = [\mathrm{EC1}/\mathrm{EC2}]\ \mathrm{X}\ 100\\ \mathrm{MSI}\ (\%) = [1 - (\mathrm{EC1}/\mathrm{EC2})]\ \mathrm{X}\ 100\\ \mathrm{MI}\ (\%) = [1 - (\mathrm{MSIs}/\mathrm{MSIc}]\ \mathrm{X}\ 100\end{array}$$

*MSIc is membrane stability index of the control leaves and MSIs is membrane stability index of Cd-stressed leaves.

Oxidative stress was furthermore tracked by identifying its markers, hydrogen peroxide level (mg/g fwt, H_2_O_2_) in 250 mg of *T. aestivum* leaves, which was quantified at 390 nm [35]. The supernatant (0.5 mL) was added to 1 M potassium iodide (2 mL) besides 0.5 mL of 10 mM potassium phosphate buffer (pH 7.0). Malondialdehyde (MDA) (nmol/ g fwt) conc. at 532 nm was used to measure lipid peroxidation, as described by Ohkawa et al. [36] exhausting the thiobarbituric acid [TBA] reaction.

### Total antioxidant capacity (TAC) in *T. aestivum* under Cd stress

After mixing the methanolic extract with a reagent solution [4 mM ammonium molybdate, 0.6 M sulphuric acid, and 28 mM sodium phosphate], the TAC content in *T. aestivum* leaves was expressed as mg/g fwt [37]. The tubes were cooled after being incubated in a boiling water bath for 90 min, and the absorbance was measured.

### Osmoprotectants in *T. aestivum* under Cd stress

Using the Bates et al. [38] method, the proline content (µmols g^− 1^ fwt), was measured in *T. aestivum* leaves fwt at 520 nm after grinding in aqueous sulphosalicylic acid (3%). After extracting the quaternary ammonium compounds from *T. aestivum* leaves using sulphuric acid (2 N) [39] and heating it to 60 °C for 10 min, Glycine betaine (GB) conc. (mg/g fwt) was measured at 356 nm. 50 µL of cold KI-I_2_ was added to 125 µL of supernatant, which was then maintained at 0 to 4 °C for 16 h before being centrifuged at 14,000 rpm for 30 min at 0 °C in order to precipitate GB as golden crystals. 1.4 mL of 1,2-dichloroethane is added to the GB crystals to dilute them. Also, the protein content in *T. aestivum* leaves fwt was assessed [40].

### Shoots and roots Cd content, TI and transfer factor (TF)

Cd content in *T. aestivum* shoot and root samples (µg/g dwt) were measured. Tri acids, which include sulphuric, nitric, and perchloric acids (3:2:1), were used to digest the samples until a clear mixture was produced. After filtering the resultant sample, using double-distilled water, the volume was adjusted. The root and shoot weights produced in Cd-contaminated soils were divided by their corresponding weights in non-contaminated soils to determine the TI of *T. aestivum*, which was then expressed as a percentage [41]. The calculation of shoot-root TF followed Gupta et al. [42].

$$\:\mathbf{T}\mathbf{F}\:=\frac{\:\:\mathrm{C}\mathrm{d}\:\mathrm{c}\mathrm{o}\mathrm{n}\mathrm{t}\mathrm{e}\mathrm{n}\mathrm{t}\:\mathrm{i}\mathrm{n}\:\mathrm{s}\mathrm{h}\mathrm{o}\mathrm{o}\mathrm{t}\mathrm{s}}{\:\:\:\mathrm{C}\mathrm{d}\:\mathrm{c}\mathrm{o}\mathrm{n}\mathrm{t}\mathrm{e}\mathrm{n}\mathrm{t}\:\mathrm{i}\mathrm{n}\:\mathrm{R}\mathrm{o}\mathrm{o}\mathrm{t}\mathrm{s}}$$

### Statistical analysis and correlation study

*T. viride* RA1 treatment had an impact on the morpho-physiological and biochemical characteristics of *T. aestivum* plants below Cd stress. To compare between the collected data, a one-way ANOVA was used. The statistical analysis was performed using SPSS software (Version 16.0, SPSS Inc., Chicago, IL, USA). The measured variables of *T. aestivum* were provided Pearson correlation coefficients. Past software (version 4.0) was used to produce principal component analysis (PCA) graphs besides hierarchical clustering analysis between the various treatments employed in this investigation. Also, data were analyzed using Two-way ANOVA (F-values) to study the impacts of *T. viride*, Cd and their interactions on the measured parameters of *T. aestivum*.

## Results

### Response of *T. viride* RA1 to Cd stress

**Fig. 1:**
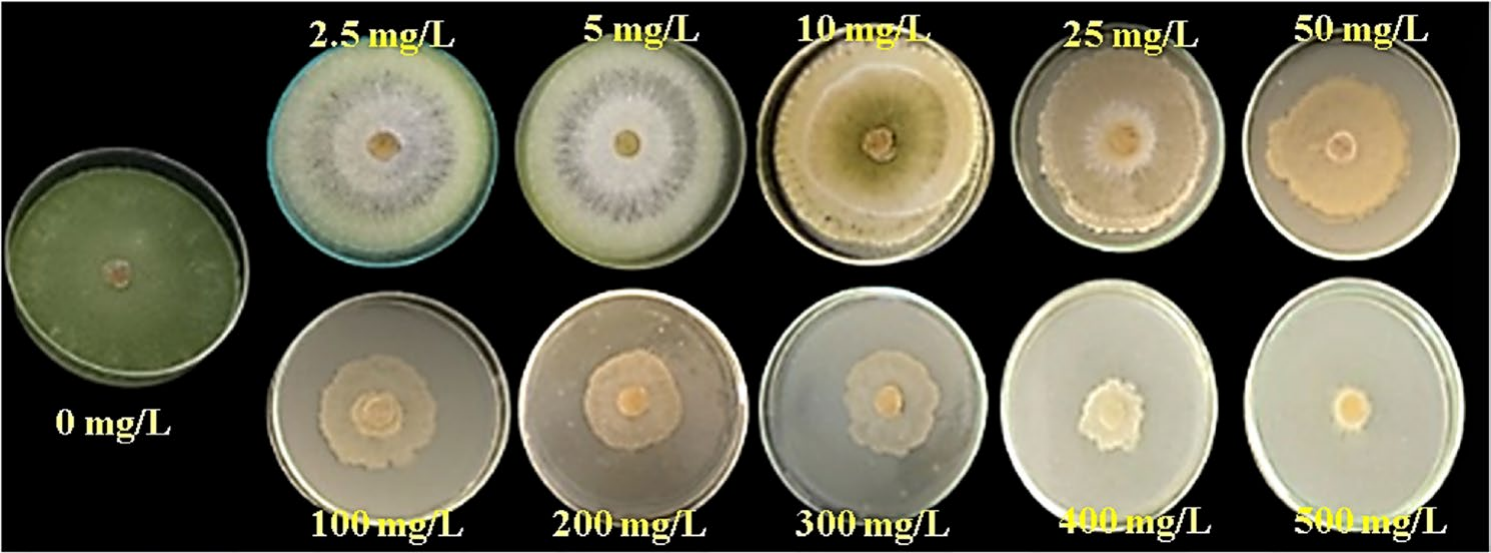
Illustrative photograph showing the growth pattern of *T. viride* RA1 fungus under different Cd conc. (mg/L) grown on the petri dishes at 27±2°C and photographed after 5 days

**Fig. 2:**
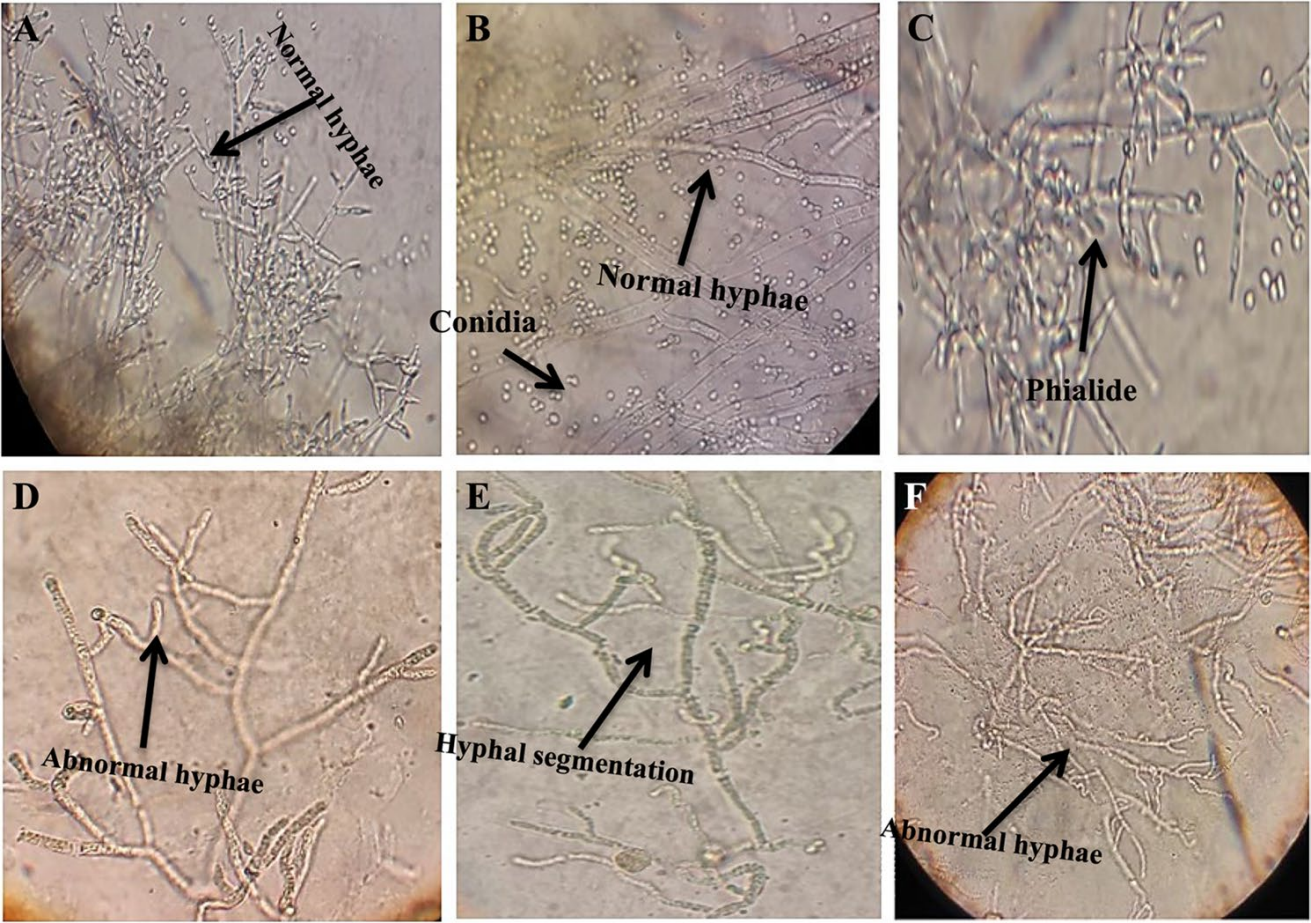
Effect of Cd stress (mg/L) on *T. viride* RA1 growth. **A**, **B** and **C** represent normal hypha (Control), while **D**, **E** and **F** represent *T. viride *hypha under Cd stress

**Fig. 3:**
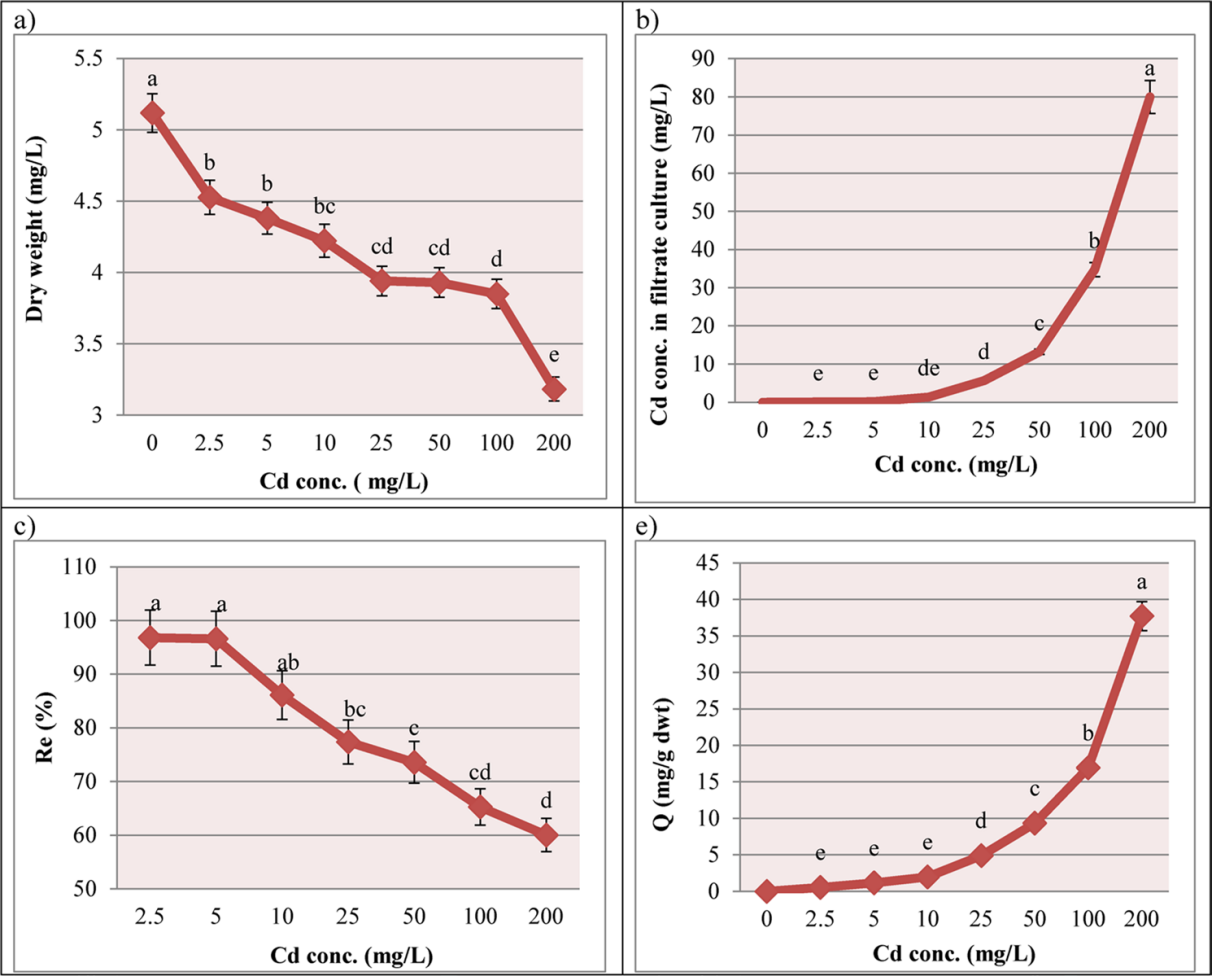
Effect of Cd conc. gradients on (**a**) the average dry weight (mg/L), (**b**) Cd conc. (mg/L) in filtrate culture, (**c**) removal efficiency (Re) and (**e**) bioaccumulation (Q) of *T. viride* RA1 mycelia grown in PDB liquid medium. *Data are mean of 5 replicates ± standard error; the dry weight was determined 7 days after inoculation. Different letters represent significant difference at the *p* ≤ 0.05 level by Duncan’s new multiple range test

To evaluate the Cd toxicity, *T. viride* RA1 was exposed to varying range of Cd (Table 2). It showed an ability to grow on different Cd conc. on PDA agar plates in the range of 0–500 mg/L (Fig. 1). The colony diameter of *T. viride* RA1 showed that the low Cd conc. (2.5, 5 and 10 mg/L) did not conspicuously affect the hyphal growth. However, the concentrations higher than 10 mg/L decrease its hyphal growth significantly (*p* ≤ 0.05) (Table 2; Fig. 1). At 5 mg/L, the *T. viride* RA1 showed whole growth like at 0 mg/L (90 ± 4.76 mm), but at 100 mg/L, the fungus was highly affected (47.33 ± 2.5 mm), and at 200 mg/L, its growth was 42 ± 2.22 mm. The highest Cd conc. (500 mg/L) decreased the growth to 12.33 ± 6.53 mm (Fig. 1). According to the microscopic examination, treating *T. viride* RA1 with a high concentration of Cd resulted in aberrant mycelial growth as well as significant morphological changes, which primarily showed up as distortion, segmentation, collapse, globular enlargements at the hyphal strand tips, and total conidium absence (Fig. 2D, E, and F). In divergence, the control treatment’s mycelia (0 mg Cd /L) are straight and well developed showing the phialides and heavily presence of conidia (Fig. 2A, B and C). Also, the changing trends in dwts of *T. viride* RA1 in liquid media are shown in Fig. 3 that stated an inverse correlation between the Cd conc. and the dwts. Concerning *T. viride* RA1 dwts, low Cd conc. (2.5 ^_^10 mg/L) cause a slight decrease in its growth (Fig. 3). Nevertheless, with higher conc., the distinction inhibitory effects can be seen.

**Table 2:** Average of colony diam. (mm) and TI (%) of *T. viride* RA1 mycelia grown in PDA medium containing different Cd conc

Cd (mg/L)	Colony diam. (mm)	Tolerance index (TI) (%)
0	90.00 ± 4.76a	0
2.5	90.00 ± 4.76a	1
5	90.00 ± 4.76a	1
10	83.00 ± 4.39a	0.922
25	72.63 ± 3.84b	0.807
50	59.33 ± 3.13c	0.659
100	47.33 ± 2.5d	0.525
200	42.00 ± 2.22d	0.466
300	37.00 ± 1.96d	0.411
400	25.83 ± 1.367e	0.287
500	12.33 ± 0.653f	0.137

*Tolerance index rating values indicate: 0.00–0.39—very low metal tolerance. 0.40–0.59—low metal tolerance. 0.60–0.79—moderate metal tolerance. 0.80–0.99—high metal tolerance. 1.00–> 1.00—very high metal tolerance

* Data are mean of 5 replicates ± standard error; the dry weight was determined 5 days after inoculation. Different letters in the same column represent significant difference at the *p* ≤ 0.05 level by Duncan’s new multiple range test

### Removal efficiency (Re) and bioaccumulation (Q) of Cd by *T. viride* RA1

The biosorption pattern of *T. viride* RA1 was expressed by Re and Q as shown in Fig. 3. The Re percentages were 96.8 ± 5.122%, 73.56 ± 3.892%, 65.25 ± 3.452% and 60.1 ± 3.175% at 2.5, 50, 100 and 200 mg/L; respectively, as it was decreased with increasing Cd conc, however the Cd uptake increased. Moreover, Fig. 3b shows the amount of the residual Cd in the broth culture that ranged between 0 and 79.78 ± 4.32 mg/L in 0 to 200 mg/L Cd conc. The value of Q amplified to some extent within the range 0 to 25 mg/L and then speedily augmented up to 200 mg/L of Cd, where the extreme extent of Q was noticed in 200 mg/L (37.706 ± 1.995 mg/g dwt). The results in Fig. 3 also displayed a negative link between the dwt of *T. viride* RA1 and the Cd uptake.

### Preliminary experiment for wheat in vitro

**Fig. 4:**
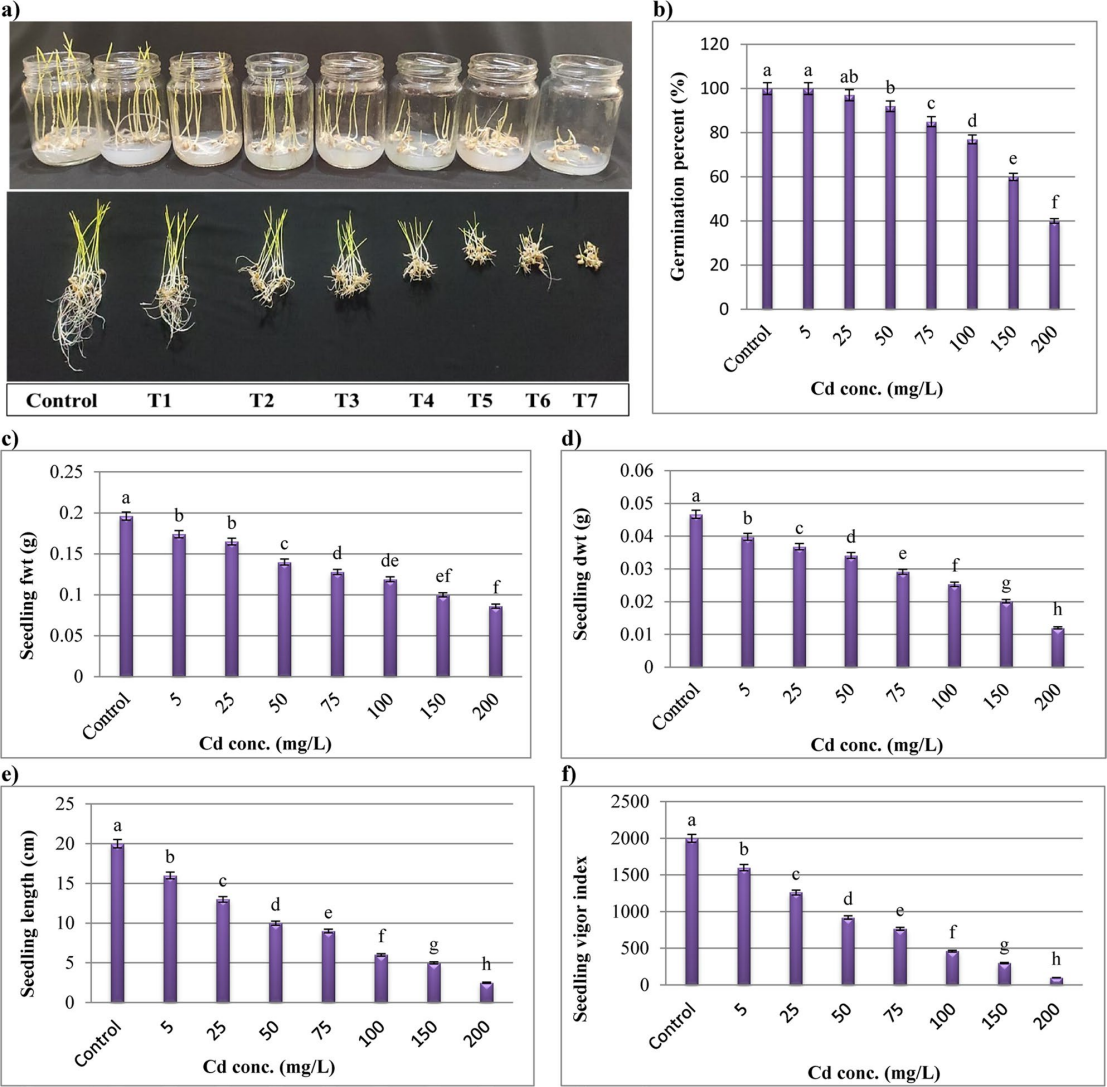
**a** Inhibitory effects of different Cd levels (mg/L) on root and coleoptile growth of wheat germinated in 0.8 water-agar; where T1-T7 are the concentrations of Cd (5, 25, 50, 75, 100, 150 and 200 mg Cd/L respectively), control represents 0 mg Cd/L. **b** Germination percent. **c** Seedling fwt. **d** Seedling dwt. **e** Seedling length and **f** Seedling vigor index. Each bar indicates means of 5 replicates ± standard error. Different alphabets on bars show significant differences among treatments

An essential first stage in life cycle of plants is seed germination. Therefore, A *T. aestivum* seedling’s in vitro growth was examined at various Cd concentrations. However, *T. aestivum* seeds were shown to be capable to germinate up to 200 mg Cd /L (Fig. 4a), despite the fact that Cd stress dramatically reduced all the seedling growth characteristics of wheat seedlings, including germination rate, seedling length, seedling vigor index, and fresh and dry mass. A significant decrease in germination percent occurred at all the levels of Cd in comparison to control (0 mg/L). Concerning Cd conc., their higher conc. caused the greatest inhibitory effects to *T. aestivum* seedlings parameters as compared to the lower ones (Fig. 4).

It was shown that the highest Cd supplementation (200 mg/L) resulted in the greatest damage and a decrease in the vigor index (95%), seedling length (87.5%), and germination percentage (60%). Additionally, compared to the control, the 200 mg Cd/L administration showed a substantial decrease in seedling fwt and dwt of 56.12% and 74.30%, respectively (Fig. 4). Therefore, Cd had a negative impact on all wheat germination parameters.

### *T. viride* RA1 alleviates Cd-induced inhibition on *T. aestivum* growth under greenhouse conditions

**Fig. 5:**
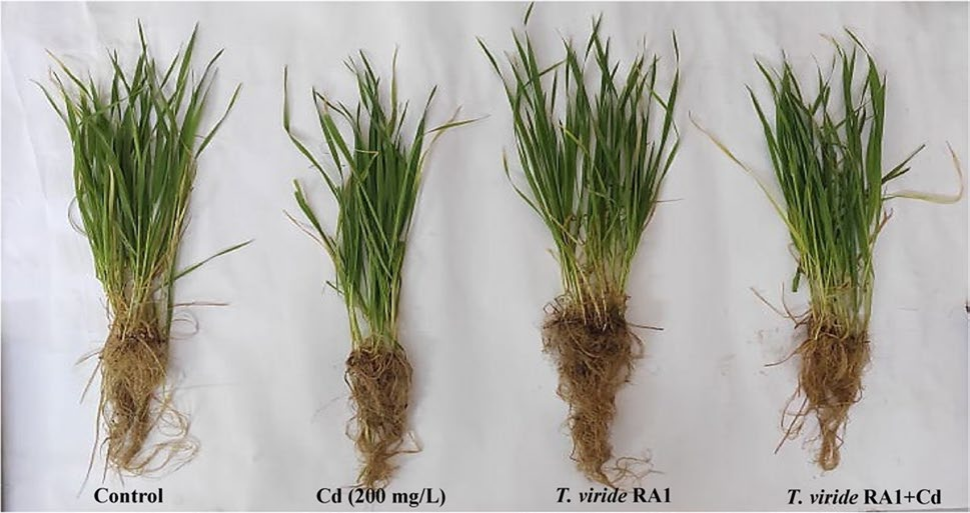
Photographs of *T. aestivum* plants showing the differences between control and Cd stressed plants under *T. viride* RA1 fungal application

The potential of *T. viride* RA1 to increase the *T. aestivum* plants tolerance to Cd (200 mg/L) was examined in a greenhouse experiment. After 30 days of Cd application, Cd had a significant inhibitory effect on fwts and dwts of *T. aestivum* as compared with control plants (Table 3; Fig. 5). On the other hand, *T. viride* RA1 outperformed non-inoculated *T. aestivum* plants in terms of growth performance under Cd stress and extending shoots and leaf number (Table 3; Fig. 5). Also, *T. viride* RA1 significantly increased the fwt and dwt of the roots by 13.9 and 16.04%, and the fwt and dwt of the shoots by 12.33 and 13.28%, respectively, when compared to the control. Thus, under control conditions, *T. viride* RA1 greatly increased *T. aestivum* growth, and under Cd stress, it dramatically dropped its negative effect on these growth metrics (14.45 and 17.49% for shoots; 44.29 and 48.78% for roots).

**Table 3:** The effect of Cd and *T. viride* RA1 application on the morphological traits of *T. aestivum*

Treatments	Shoot length (cm/plant)	Shoot fwt (g/plant)	Root fwt (g/plant)	Shoot dwt (g/plant)	Root dwt (g/plant)	Leaves number (no./plant)
Control	17.5 ± 0.463b	2.092 ± 0.055b	1.86 ± 0.049b	0.3854 ± 0.01b	0.2718 ± 0.0072b	6 ± 0.1587b
Cd (200 mg/L)	12.5 ± 0.331d	1.736 ± 0.0459c	1.095 ± 0.0289d	0.3034 ± 0.008c	0.157 ± 0.0042d	5 ± 0.1322c
*T. viride*	20 ± 0.529a	2.35 ± 0.0622a	2.12 ± 0.056a	0.4366 ± 0.0115a	0.3154 ± 0.0083a	7 ± 0.185a
*T. viride* + Cd	14 ± 0.37c	1.98 ± 0.051c	1.58 ± 0.0418c	0.356 ± 0.0094b	0.2336 ± 0.0062c	6 ± 0.158b

Different letters in the same column represent significant difference at the *p* ≤ 0.05 level by Duncan’s new multiple range test

* Data are mean of 5 replicates ± standard error

### *T. viride *RA1 alleviates Cd-induced inhibition of photosynthetic pigments

We measured the photosynthetic parameters of *T. aestivum* leaves, such as Chl a, Chl b, carotenoids, and total pigment, following a 200 mg Cd/L application with the intention of investigating the effects of *T. viride* RA1 inoculation on photosynthesis. Cd exposure dramatically decreased Chl a and Chl b by 47.30 and 41.79%, while *T. viride* RA1 inoculation alleviated Cd toxicity by decreasing this reduction (24.88% for Chl a and 22.15% for Chl b as compared to the control) (Table 4). Besides Chl a and Chl b, carotenoids and total pigment decreased (42.64 and 44.66%) under Cd stress compared to control. Despite the fact that *T. viride* RA1 inoculation increased photosynthesis in *T. aestivum* leaves, the results were noticeably greater than those obtained with Cd alone (Table 4). The *T. aestivum* plants that had been treated to *T. viride* RA1 showed the maximum rise. In addition to considerably reducing the Cd-induced decrease in leaf photosynthesis, this can lead to an increase in chlorophyll and other supplemental pigments in the plant leaves.

**Table 4:** Role of Cd tolerant *T. viride* RA1 on leaf pigments in *T. aestivum* (L.) cultivated in Cd- stressed soil

Treatments	Chl a (mg/g fwt)	Chl b (mg/g fwt)	Chl a + b (mg/g fwt)	Carotenoids (mg/g fwt)	Total pigment (mg/g fwt)
Control	1.503 ± 0.0397b	0.713 ± 0.0188a	2.216 ± 0.0586b	0.945 ± 0.025b	3.161 ± 0.084b
Cd (200 mg/L)	0.792 ± 0.021d	0.415 ± 0.0109c	1.207 ± 0.0319d	0.542 ± 0.014d	1.749 ± 0.046d
*T. viride*	1.665 ± 0.044a	0.762 ± 0.02a	2.427 ± 0.064a	1.051 ± 0.028a	3.477 ± 0.092a
*T. viride* + Cd	1.129 ± 0.0298c	0.555 ± 0.0147b	1.684 ± 0.044c	0.728 ± 0.019c	2.413 ± 0.064c

Different letters in the same column represent significant difference at the *p* ≤ 0.05 level by Duncan’s new multiple range test

*Data are mean of 5 replicates ± standard error

### Alleviation of stress markers induced by Cd stress by *T. viride* RA1

In *T. aestivum* leaves, 200 mg Cd/L caused a substantial decrease in MSI by 16.15% and increased EL and MI by 51.33 and 16.15% in comparison with the control (Table 5). Besides, Cd caused a substantial increase in H_2_O_2_ manufacture (32.22%) resultant in excessive ROS making and ultimately increased the MDA content (67.74%). MDA content and EL% of *T. aestivum* leaves increased, which would explain the decline in MSI (Table 5).

**Table 5:** Role of Cd tolerant *T. viride* RA1 fungal application on oxidative stress biomarkers of *T. aestivum* (L.) leaves cultivated in Cd- stressed soil

Treatments	MSI %	EL %	MI %	MDA (nmol/g fwt)	H_2_O_2_ (mg/g fwt)
Control	76.059 ± 2.0123b	23.941 ± 0.633b	-	2.48 ± 0.114c	8.41 ± 0.223c
Cd (200 mg/L)	63.768 ± 1.687c	36.232 ± 0.958a	16.159	4.16 ± 0.191a	11.12 ± 0.294a
*T. viride*	84.615 ± 2.2387a	15.385 ± 0.407c	-	2.54 ± 0.116c	6.34 ± 0.167d
*T. viride* + Cd	74.790 ± 1.978b	25.210 ± 0.667b	11.613	3.29 ± 0.151b	10.04 ± 0.266b

*MSI* Membrane stability index, *EL* Electrolyte leakage, *MI* Membrane injury and *MDA* Malondialdehyde

* Data are mean of 5 replicates ± standard error. Different letters in the same column represent significant difference at the *p* ≤ 0.05 level by Duncan’s new multiple range test

To increase the performance of membranes and ROS scavenging in leaves, it is necessary to inoculate *T. aestivum* with *T. viride* RA1 to minimize the Cd negative impacts. Since *T. viride* RA1 considerably reduced the negative effects of Cd, our investigation demonstrated that all oxidative stress biomarkers were comparatively low when applied to Cd-stressed plants. Accordingly, the most noticeable decrease in MDA, EL and H_2_O_2_ was noted in *T. viride* RA1-treated plants with approximately 20.91, 30.42 and 9.71%, resulting in statistically significant cellular protection compared to Cd-stressed *T. aestivum* plants (Table 5).

### *T. viride* RA1 increases the TAC in *T. aestivum* under Cd stress

**Fig. 6:**
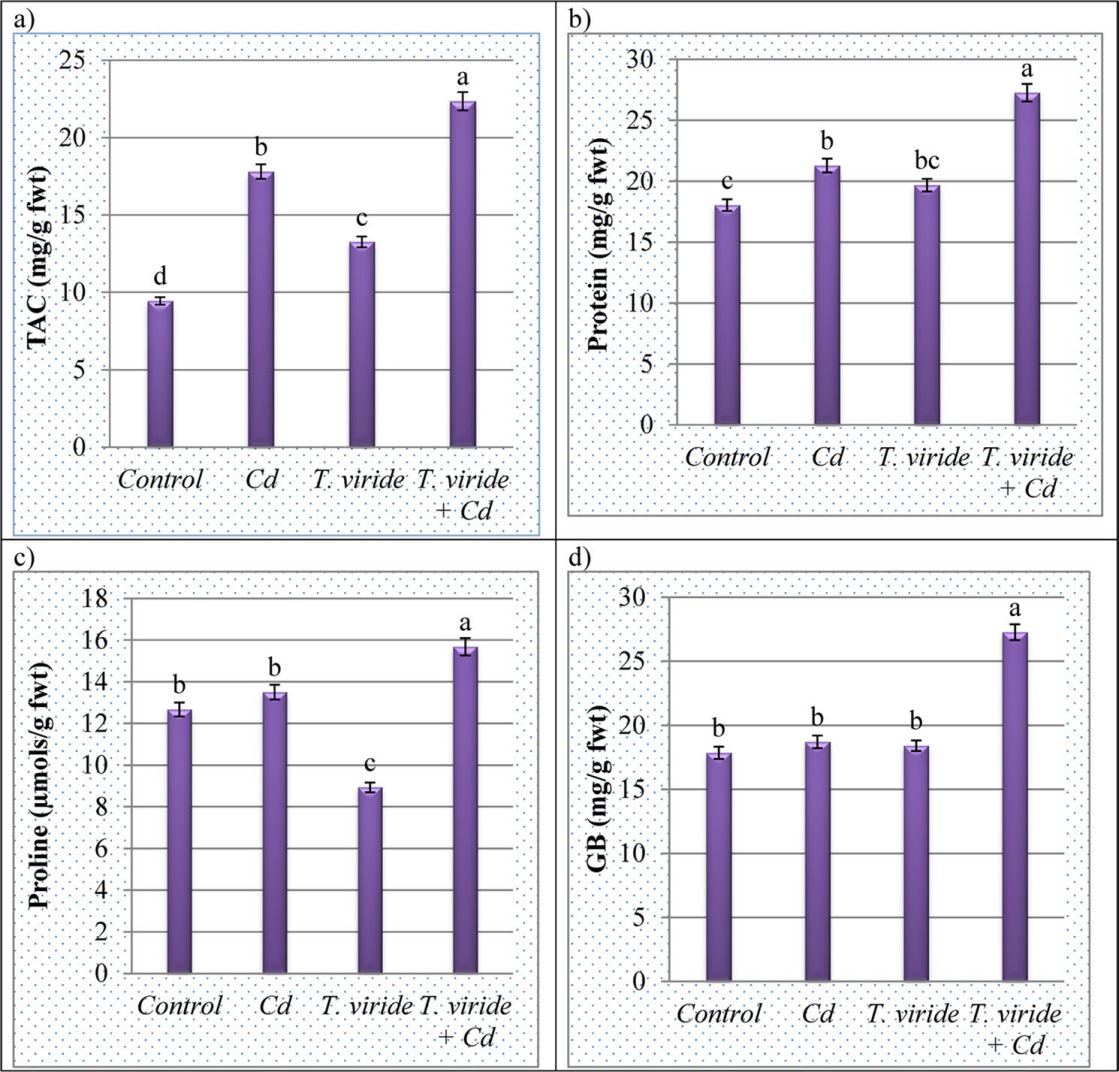
The effect of 200 mg Cd/L and *T. viride* RA1 application on the osmoprotectants content of *T. aestivum* plant leaves. * Data are mean of 5 replicates ± standard error. Different letters in the same column represent significant difference at the *p* ≤ 0.05 level by Duncan’s new multiple range test

In our study, in reaction to oxidative damage (MDA, EL, MI and H_2_O_2_) as previously mentioned, *T. aestivum* plants create various antioxidant enzymes and osmoprotectants to safeguard themselves from the destructive effects of Cd. Figure 6 indicated that the TAC was seriously increased by 200 mg Cd/L stress imposed in *T. aestivum*. Under Cd stress, *T. aestivum* leaves had higher values of TAC (17.80 ± 0.471 mg/g fwt) than those under controlled conditions (9.44 ± 0.249 mg/g fwt) (Fig. 6). However, the highest TAC content was reported in *T. aestivum* leaves treated with *T. viride* RA1 (22.35 ± 0.591 mg/g fwt).

### *T. viride* RA1 increases osmoprotectants contents in *T. aestivum* under Cd stress

**Fig. 7:**
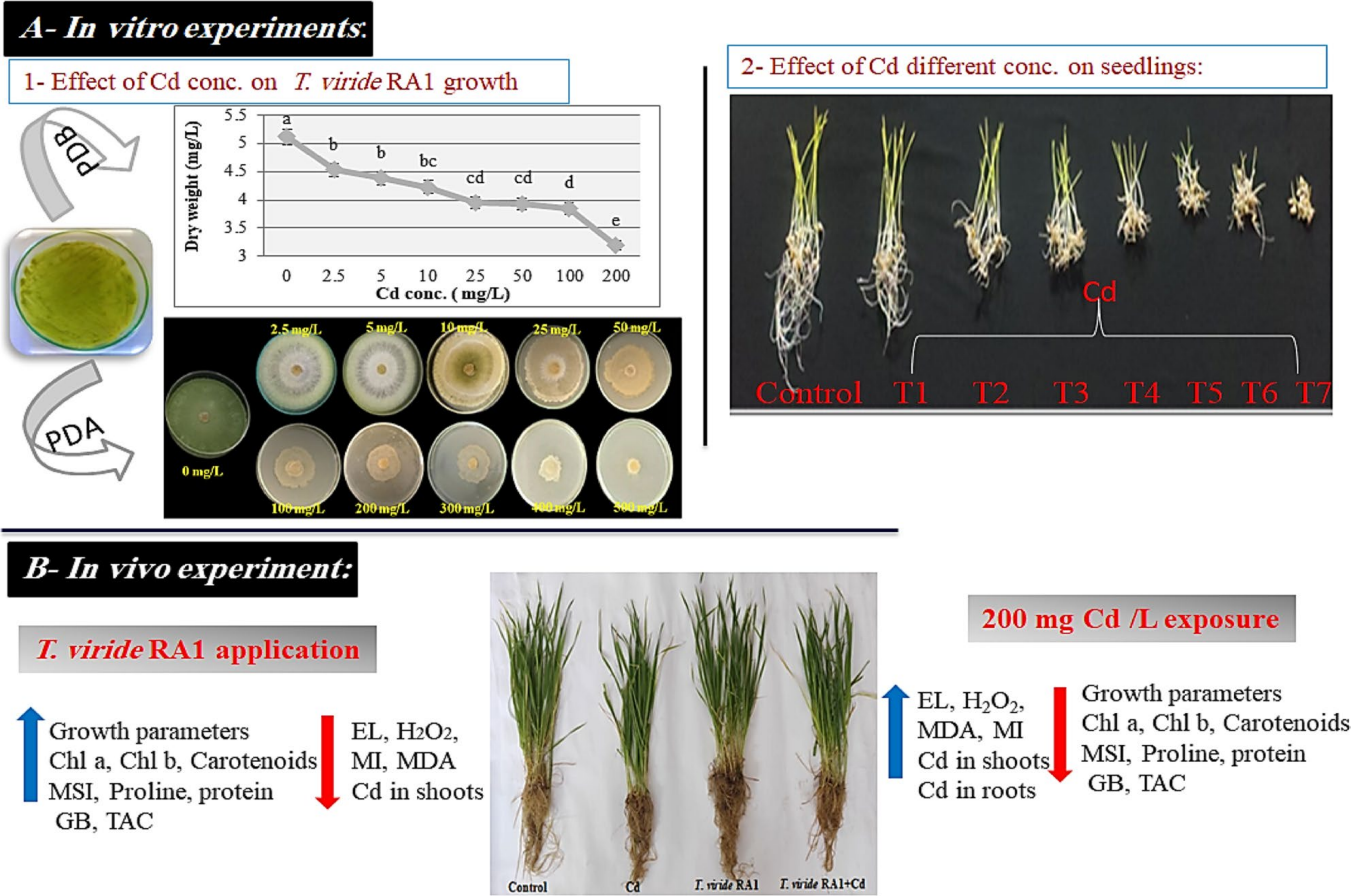
Schematic overview and key results of *T. aestivum* plants after treatment with Cd and *T. viride* RA1 (discussed in detail in the text). Increase is indicated in blue arrow and decrease is indicated in red arrow

After *T. aestivum* plants exposure to 200 mg Cd/L stress, their leaves accumulated a substantial amount of osmoprotectants, such as soluble proteins, GB, and proline contents, in comparison to the control. Additionally, *T. viride* RA1 application maximized their contents. The highest increase in proline content (16.14%), GB (24.95%) and protein (28.07%) was observed in *T. aestivum* plants treated with *T. viride* RA1 under Cd imposition compared to the Cd- stressed *T. aestivum* plants. Figure 6 showed that *T. viride* RA1 effectively counteracted the negative effects of Cd stress and resulted in a notable rise in all osmoregulatory components as compared to the control and Cd-stressed *T. aestivum* plants as also represented in Fig. 7 which shows a schematic overview of the key results of *T. aestivum* plants after treatment with Cd and *T. viride* RA1.

### *T. viride* RA1 decreases Cd translocation to *T. aestivum* shoots

**Fig. 8:**
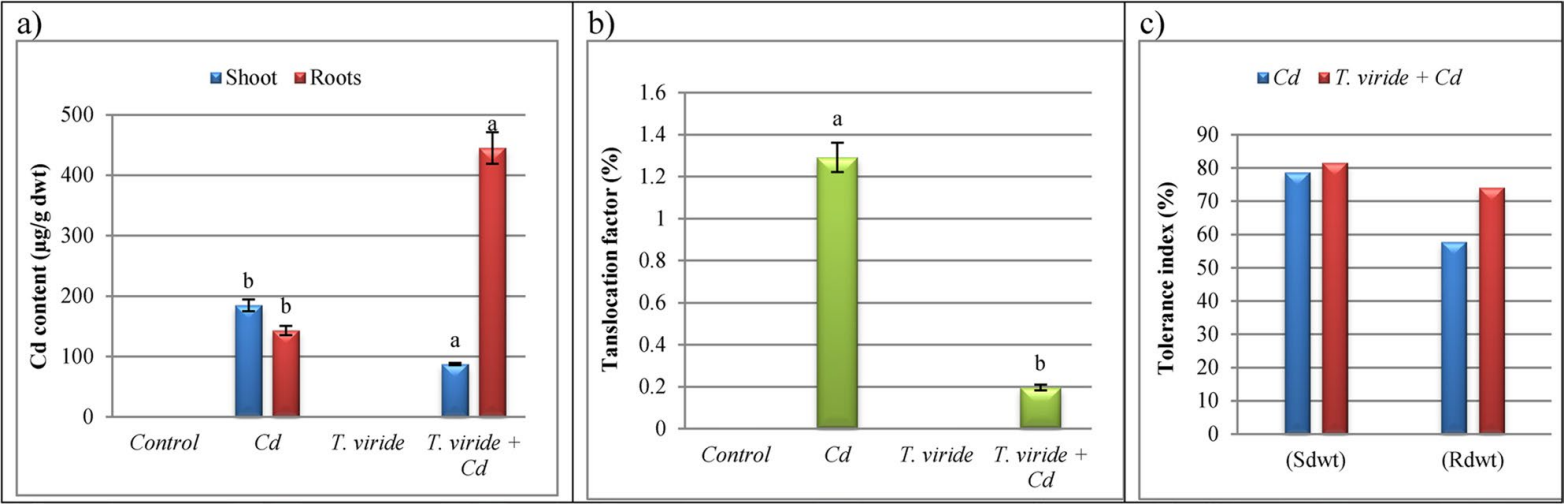
Cd content in shoots and roots (**a**), translocation factor (TF) (**b**) and tolerance index (TI) (**c**) of *T. aestivum* plant leaves upon 200 mg Cd/L and *T. viride* RA1 application.* Data are mean of 5 replicates ± standard error. Different letters represent significant difference at the *p* ≤ 0.05 level by Duncan’s new multiple range test

Our data showed that the Cd in *T. aestivum* shoots and roots was significantly increased under Cd stress and its content was higher in *T. aestivum* roots (143.25 ± 7.58b µg/g dwt) and shoots (185 ± 9.78 µg/g dwt) compared to the non-stressed ones (Fig. 8), indicating that Cd is confined in the roots and subsequently transported to the shoots. Captivatingly, following inoculation of metal-tolerant *T. viride* RA1 to *T. aestivum* plants, Cd accumulation were significantly increased in their roots (445.25 ± 26.21 µg/g dwt) and reduced in their shoots (87.5 ± 1.98 µg/g dwt) by 210.82 and 52.70%, respectively, over non-inoculated ones. Concurrently, when Cd was applied, *T. aestivum’s* TF rose, resulting in more HMs being transferred from roots to shoots (1.291 ± 0.07) (Fig. 8). However, following *T. viride* RA1 inoculation, this factor was substantially decreased (0.196 ± 0.0138).

### Correlation study

**Fig. 9:**
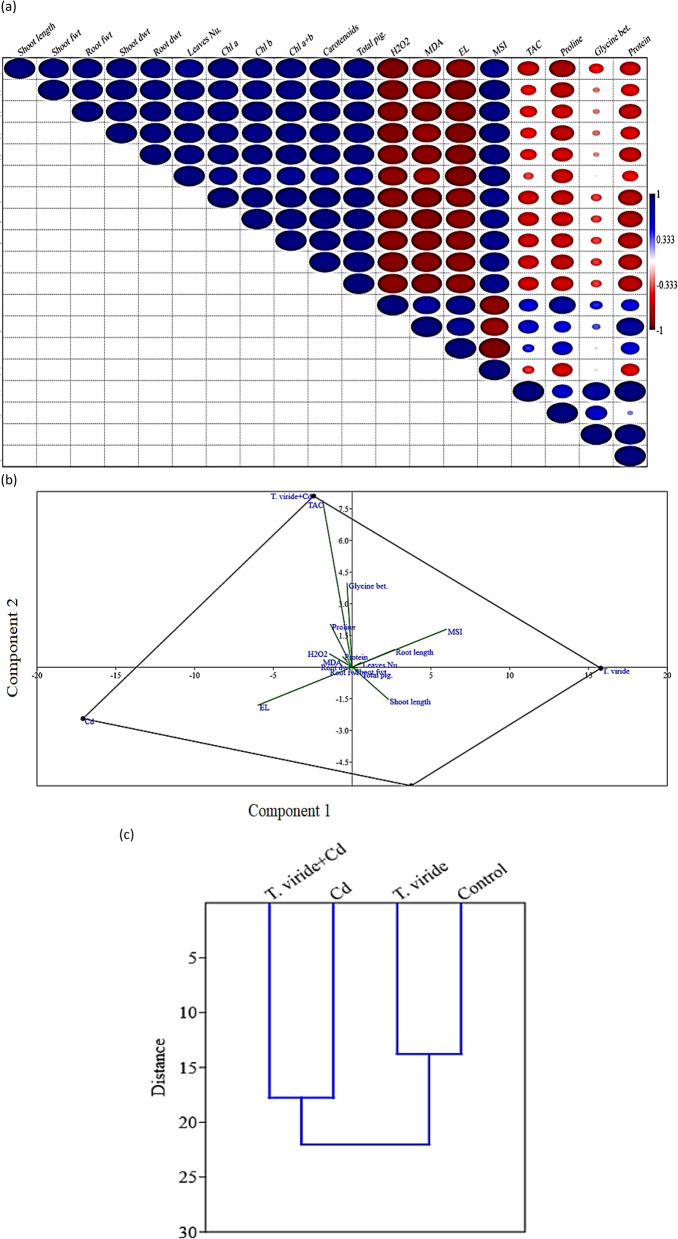
**a** Correlation analysis among the studied parameters. Blue in the box indicates significantly positive correlations, while red in the box indicates significantly negative correlations. **b** The PCA was applied to study the interaction between variables (parameters studied) and observation (treatments), and **c** Hierarchical clustering analysis

To comprehend the relationships between *T. viride* RA1 treatment and the various morpho-physiological as well as biochemical parameters in *T. aestivum* below Cd stress, PCA and Pearson’s correlation analysis were performed. Additionally, Pearson’s correlation matrix indicates a substantial association between the various morpho-physiological traits as illustrated in Fig. 9; Table 6. In conclusion, there were favorable relationships between the growth parameters and plant height, fwt, dwt, and chlorophyll content. On the other hand, they exhibited unfavorable associations with H_2_O_2_ levels and lipid peroxidation.

**Table 6:** Two-way ANOVA analysis (F-values) of *T. viride*, Cd and their interactions on the measured parameters

Parameters	*T. viride*	Cd (200 mg/L)	*T. viride* + Cd
Shoot length	21.49***	163.3***	1.35ns
Shoot fwt	14.70**	57.7***	0.937ns
Root fwt	68.06***	208.8***	6.21*
Shoot fwt	27.58***	67.69***	0.005ns
Root fwt	81.72***	218.6***	6.16*
Leaves number	39.14***	39.139***	0.0ns
Chl. a	51.31***	320.41***	6.3*
Chl. b	32.52***	232.16***	7.54*
Chl. a + b	44.80***	290.5***	6.69*
Carotenoids	43.17***	266.88***	3.241ns
Total pigments	44.30***	282.8***	5.59*
MSI	24.22***	30.9***	0.384ns
EL	198.60***	253.5***	3.135ns
MDA	23.01***	207.1***	30.33***
H_2_O_2_	42.27***	175.02***	4.175*
Proline	5.20*	122.8***	74.9***
Protein	43.50***	88***	14.1**
TAC	92.30***	402.4***	0.706ns
GB	25.14***	31.14***	15.5**

Significance levels: *P ≤ 0.05; **P ≤ 0.01; ***P ≤ 0.001; ns, non-significant effect

## Discussion

Cd is mostly found in the environment due to industrial contamination and has harmful effects on life. Certain microorganisms, such as the biocontrol fungus *Trichoderma* spp, can live in contaminated environments and function as bioremediators [43]. Regarding the response of *T. viride* RA1 fungal growth to Cd stress; low concentrations of Cd did not affect the hyphal growth of *T. viride* RA1. However, the higher concentrations decrease its hyphal growth and sporulation. Thus, the inhibitory effect of Cd appeared to be a concentration-dependent as El-Sayed et al. [44] reported in *A. flavus* MT639638 upon exposure to Fe conc. Also, *Pleurotus* spp. was repressed by high Cd conc. as Li et al. [41] described. The inverse correlation between the Cd conc. and the dwts, showed in this study, was conforming with He et al. [17] who conveyed that with increasing Cd conc., the dwt of *Penicillium funiculosum*, *P. aurantiogriseum* and *Alternaria tenuissima* showed a decreasing trend. Metwally et al. [7] investigated the inhibition of *A. niger* and *A. terreus* growth at 200 mg/L Cd. Likewise, Maurya et al. [4] found that Cd besides Pb caused destructive effects on biomass as well as sporulation of *T. asperellum* and *Hypocrea nigricans* at higher concentrations. The growth inhibition of Cd may be attributable to ROS production from the Fenton reaction [3, 17, 45]. Moreover, *T. viride* RA1 showed a high tolerance at Cd conc. (0–25 mg/L); however this tolerance decreased at higher Cd conc. indicating its inhibitory effect at higher conc. Therefore, this fungus can be utilized as a biological adsorbent and remediator of Cd contamination since it is tolerant to Cd.

Conformity with biosorption data, Abeed et al. [46] reported that *T. harzianum* has a great tolerance to varying Cd conc. (0–300 mg/L) and can absorb large amounts of Cd to achieve 90% removal efficiency. Moreover, the finding of *T. viride* RA1 potentiality for Cd removal was in accordance with Yaghoubian et al. [47]. According to Mohsenzade and Shahrokhi [48], Cd absorption by *T. harzianum* ranged from 1 to 100 mg/L. Previous studies revealed that the HM accumulation on fungal cell wall surfaces was a major factor in its uptake by fungi [21, 47]. Therefore, it was noted that the cell wall fraction contained 79.0% of the Cu that *T. viride* had gathered [21]. Furthermore, according to Lopez Errasquın and Vazquez [49], *T. atroviride’s* uptake of metals was due to physical attachment to cell surfaces rather than a metabolically reliant mechanism. Metal binding to microbial biomass, biosorption and bioaccumulation mechanisms [46, 50] can be used to explain *Trichoderma’s* tolerance to Cd. Strategies for HMs biosorption by fungi include surface adsorption, micro-precipitation, ion chelation besides ion exchange [51]. Kapoor and Bhatnagar [52] supported this theory by showing that fungal mycelia have a high capacity for metal sorption as mycelia reduce their harmful effects and hence limit their transfer to plants by keeping metals inside their fungal structure [53], . Furthermore, functional groups comprising carboxyl, amino, hydroxyl, as well as phosphate that found on the surface of fungal cells are essential for the Cd ions uptake [51].

Seed germination is a crucial first phase in the life cycle of plants. This study revealed that all of the wheat germination characteristics were negatively impacted by Cd. Similarly, Cd conc. reduced the germination %, germination index, vigor index, and seedling length of *Pinus massoniana* and *V. radiata* [3, 54]. The presence of a hazardous HMs in the germination media is the cause of the decline in seed germination as Kuriakose and Prasad [55] reported a similar decrease in sorghum seed germination when exposed to Cd due to the decline in physiological and metabolic activities, for example a decreased flow of water and reserves on the way to the embryonic axis [3, 56]. Furthermore, Rahoui et al. [57] showed delays in germination of pea and faba bean seeds with Cd stress as a result of membrane degradation and solute leakage. Furthermore, the significant drop in fwt and dwt of *T. aestivum* seedlings may be attributable to a grouping of factors, including decreased PSII activity, inhibition of cell division, and direct or indirect regulation of numerous physiological processes similar photosynthesis, respiration, and the plant-water relationship. In conclusion, these elements reduce cell activity, which hinders plant biomass and growth [56].

Regarding the greenhouse experiment, a concentration of 200 mg L^− 1^ of Cd was used to induce a consistent and severe level of metal stress in controlled pot conditions, allowing for a clear evaluation of stress-mitigation mechanisms. Additionally, it is frequently employed in mechanical studies to mimic highly contaminated soils in order to elucidate microbial-mediated tolerance responses in plants, thus evaluating *T. viride* RA1’s capacity to reduce Cd stress rather than to replicate typical agricultural soils. Our results of wheat growth inhibition comply with Deng et al. [58] and Zulfiqar et al. [59] in maize as well as wheat under Cd stress. Moreover, Abdelhameed and Metwally [1], Deng et al. [58] and Zhang et al. [60] stated that plant growth and development were negatively impacted by Cd stress, which poses a serious threat to agricultural productivity. According to Abeed et al. [46]. and Eissa and Abeed [61], excessive concentrations of HMs cause plant death by interfering with a number of biochemical processes, such as respiration, transpiration rates, nutrient uptake, cell elongation, hormone balance by changing water relations, which worsens the metal-induced growth reduction, and by lowering the endogenous level of the growth-promoting hormone auxin, which is brought on by increased activity of the auxin-degrading enzymes.

The promoter impact of *T. viride* RA1 was noticeable on wheat growth indicating its bio-protective effect. The low bioavailable Cd content may also be the cause of its beneficial effect on wheat plant growth. Abeed et al. [46] shown that soil containing up to 150 mg of Cd/kg was required for *T. harzianum* to have a significant stimulating effect on sunflower fwt and dwt. After inoculating *Brassica juncea* (L.) and *Vigna radiata* (L.) exposed to Ni, Pb, and Cd stress with *T. atroviride* and *Trichoderma* spp. TF-13, Altaf et al. [3] and Cao et al. [62] conveyed a significant improvement in morphological features. This suggests that *T. viride* RA1 can withstand Cd and continue to function even at lethal doses for the plant. These results imply that *T. viride* RA1 inoculation reduced Cd toxicity due to its metal detoxification ability.

Concerning the photosynthetic pigments, their decline in *T. aestivum* leaves with Cd lines up perfectly with Altaf et al. [3] and Zhang et al. [60] in *V. radiata* as well as *Nicotiana tabacum* owing to its damaging effect on chloroplast structure and reducing the chlorophyll synthesis by heightening chlorophyllase activity, degrading chlorophyll molecules, and substituting the central Mg^2+^ in the porphyrin ring [63, 64]. Furthermore, the generation of ROS consequently of Cd toxicity causes shrinkage in pigment levels that leads photo-oxidative damage and photo-inhibition in *T. aestivum*’s chloroplasts. Additionally, Cd ions in the chloroplast affect the Calvin cycle, changing the rate of photosynthesis and slowing down the photosynthetic pigments [3, 28].

Our findings of increasing photosynthetic pigments with *T. viride* RA1 are align with Oljira et al. [65], who found that *T. yunnanense* and *T. afroharzianum* enriched water use efficiency, plant biomass, and photosynthesis underneath stress. Additionally, under HMs stress, inoculation with *Trichoderma* spp. TF-13 and *T. harzianum* effectively improves the leaf pigment features of tomato and *V. radiata*, according to reports by Altaf et al. [3] and Vukelić et al. [66]. Our study exhibited that *T. viride* RA1-treated plants had higher levels of osmolytes such as proline, GB, and protein, which may help to stimulate the development and photosynthesis of *T. aestivum* plants exposed to Cd.

When plants absorb excessive amounts of HMs, there is an imbalance in the free radicals produced and scavenged within cells. This leads to ROS accumulation of large amounts, which extra cause the peroxidation of unsaturated fatty acids in membranes, damaging their structure and function and a loss of their integrity [2, 46, 67, 68]. Ahanger and Agarwal [69] documented that under environmental stressors; significant intensities of oxidative biomarkers for instance EL, MDA, and H_2_O_2_ were elevated that they impair photosynthetic efficiency, Chl. conc., and membrane stability [70]. Our results of stress markers are in line with Jia et al. [71] who reported that Cd stress markedly boosted the increase of EL in the cabbage seedlings due to the significant ROS production. From a physiological point of view, the elevated intensities of EL, MDA, and H_2_O_2_ in *T. aestivum* plants have been linked to the enhanced dehydration of cytoplasm and breakdown of plasma membranes brought on by Cd stress. Our findings demonstrated that Cd markedly boosted the production of MDA, suggesting elevated lipid peroxidation, excessive ROS buildup, severe cellular redox imbalance and oxidative damage [72].

*T. viride* RA1’s beneficial effects on *T. aestivum* plants in reducing oxidative damage seem to be connected to the amplified activity of antioxidants through the speedy elimination of H_2_O_2_ brought on by Cd, which in turn supports and encourages plant growth. Our findings prove the observational data with Sofy et al. [73]. Besides, Metwally and Soliman [18] explained that *T. viride* reduces H_2_O_2_ accumulation in tomato seedlings under salt stress and improved membrane permeability indicating their cellular free radical quenching system is more effective. Additionally, Altaf et al. [3] reported that, in comparison to uninoculated *V. radiata*, *Trichoderma* spp. TF-13 inoculation reduced the toxicity and effectively declined the levels of H_2_O_2_ and MDA in Cd-stressed *V. radiata*. Additionally, *Trichoderma* spp. may have a part in maintaining intracellular water relations below stress [73, 74], since biomass accumulation has significantly improved as a result of increased water intake and metabolite production.

The oxidative stress caused by Cd harms the cellular membrane besides electron transport, inhibits or activates enzymes, and interferes with photosynthesis as well as nucleic acids, all of which lead to growth delay [75]. As consequences, plants have advanced a defensive mechanism against ROS accumulation, which involves the production of osmoprotectants as well as antioxidant system activation. Ascorbic acid and glutathione are examples of non-enzymatic antioxidants, while superoxide dismutase, catalase, ascorbate peroxidase, and glutathione-S-transferase are examples of enzymatic antioxidants [18, 75]. In plant cells, all of these antioxidants effectively scavenge H_2_O_2_. Zhang et al. [76] stated that Cd stress increased peroxidase, and ascorbate peroxidase activities in *Canna orchioides* leaves. Also, Altaf et al. [3] reported that enzymatic and non-enzymatic (proline, catalase, peroxidase, superoxide dismutase, and glutathione reductase) activities of *Trichoderma*-inoculated in Cd and Pb-treated plants were enhanced. In line with our research, mycorrhizal fungal inoculation altered fenugreek plants’ resistance to Cr stress by controlling proline metabolism; in this case, it significantly reduced metal stress by reducing stress biomarker levels [28]. Furthermore, a study asserted that exposing plants to beneficial fungi enhanced their tolerance to HMs in *T. aestivum* L [77].

In addition to increasing TAC, a marked increase in the osmolytes (soluble proteins, GB, and proline) was observed with Cd and *T. viride* RA1 application. Our findings are consistent with Akhtar et al. [78], who revealed that wastewater led to elevated GB and proline levels in wastewater-treated leaf, shoot, and root of rice plants. According to Roychoudhury and Banerjee [79] and Jogawat [80], in order to preserve homeostasis within a cell or in the surrounding fluid, these osmolytes are overproduced and stockpiled during osmotic stress that helps the cells to become tolerant and protects them from the damaging effects of like HMs. They also serve as antioxidants, osmoprotectants, photosynthetic protectors, macromolecule stabilizers, protein folding enhancers, and ROS detoxifiers [81]. Hare et al. [82] stated that the production of proline and GB influences the distribution of photo-assimilate between the roots and the shoots tissue and buffers the cellular redox potential, as well as polyamines such as proline that play crucial roles in defending macromolecules including DNA, protein, and enzymes [2, 80, 83, 84].

Our results of increasing osmoregulatory components with RA1 application are consistent with those of Metwally and Soliman [18], who found that *T. viride* raised the levels of protein and proline in tomato plants under salinity stress in comparison to non-inoculated plants. Nevertheless according to Altaf et al. [3], inoculating metal-stressed *V. radiata* with the *Trichoderma* spp. TF-13 reduced the amount of proline and lessened the toxicity of Cd. Akhtar et al. [78] also reported that the seeds priming with *Bacillus cereus* and *L. macroides* decreased proline and GB contents in rice plants grown in contaminated water.

Plants as well as humans are seriously threatened by the easy uptake of Cd from the soil by their roots, which can then accumulated in their aerial plant parts, including edible parts [85, 86]. Cd^2+^ absorption as well as translocation can be facilitated by a variety of transporters which are in charge of Mn^2+^, Cu^2+^, and Zn^2+^. Thus, more Cd absorption increased the concentrations of Zn, Fe, Mg, and Mn in Harukaze roots, indicating that the metal transport systems of the roots had a similar affinity for Cd for those essential divalent elements [87]. Align with our results of Cd uptake; Abeed et al. [46] and Soliman and Abdelhameed [88] revealed an increase in roots and shoots metal uptake of *V. radiata* and *Vicia faba* with increasing Cd and Pb dosages.

*Trichoderma* spp. can be a supportive bioremediation microbe that helps plant absorb HMs, lowering Cd conc. in crops [89, 90]. By retaining HMs *via* chelation on melanin-like polymers or adsorption to cell walls or immobilization by the production of insoluble metal oxalate, fungal mycelia can lower the percentage of Cd bioavailability in the rooting Sect [91]. So, the amount of metal that translocated to the *T. aestivum* shoots will be reduced. According to Wang et al. [92], soybeans inoculated with *Curvularia lunata* showed a substantial decrease in both TF and Cd content. By reducing the metal accumulation in plant organs, *T. atroviride* dramatically increased the phytoextraction efficiency of *B. juncea* exposed to Ni and Cd [62]. Conflicting with our results, Altaf et al. [3] stated that Cd absorption by *V. radiata* roots inoculated with *T. harzianum* was reduced over non-inoculated plants.

Since these rhizospheric microorganisms limit the movement of metals through the incorporation of cell-surface polymeric molecules. Our finding implies that *T. viride* RA1 application decreased the Cd translocation in *T. aestivum* shoots by trapping it in their roots. Because the metal-tolerant fungus accumulated nearly all of the metal found in the soil, *V. radiata* inoculated with this fungal isolate showed less metal deposition in their aerial parts [3]. As a result, the amount of Cd that plants could translocate was limited. Thus, *T. viride* RA1-inoculated *T. aestivum* had a higher probability of surviving even in Cd-contaminated soil as a result of the metal pressure being relieved and it also suggested its potential role in bioremediation.

### Limitations and prospective application

The present study highlights how Cd hurts *T. aestivum* growth as it is readily taken up and absorbed in their shoots and roots causing comparable changes in the physio-biochemical responses and reduction in vegetative growth parameters. However, *T. viride* RA1 may mitigate Cd stress in *T. aestivum* by improving its morphology, physiological functions, and biochemistry. However, direct field extrapolation is constrained by the use of unrealistic Cd concentrations and the lack of molecular investigations. Thus, genetic engineering may be a useful technique in the future for reducing HM stresses in sustainable agriculture by introducing genes from HMs-resistant microorganisms. Also, it should be pointed out that the study’s 200 mg L^− 1^ Cd concentration is higher than the usual background levels in the majority of agricultural soils to accurately understand the protective processes mediated by *T. viride* RA1 under controlled conditions. Although this approach improves mechanistic understanding, consideration should be utilized when extrapolating directly to field settings. Therefore, additional research using field-based trials and environmentally relevant Cd concentrations is needed to confirm the practical application of the current findings. Notwithstanding these limitations, the findings imply that *T. viride* RA1 could be used as a biostimulant to improve wheat resistance in contaminated soils, providing a promising foundation for further studies on sustainable, microbe-assisted heavy metal mitigation approaches for agriculture.

## Data Availability

The relevant datasets supporting the results of this article are included within the article.

## Abbreviations

EL: Electrolyte leakage
dwt: Dry weights
fwt: fresh weight
GB: Glycine betaine
MDA: Malondialdehyde
MSI: Membrane stability index
MI: Membrane injury
Re: Removal efficiency
ROS: Reactive oxygen species
TI: Tolerance index
TF: Translocation factor
TAC: Total antioxidant capacity
Q: Bioaccumulation capacity
